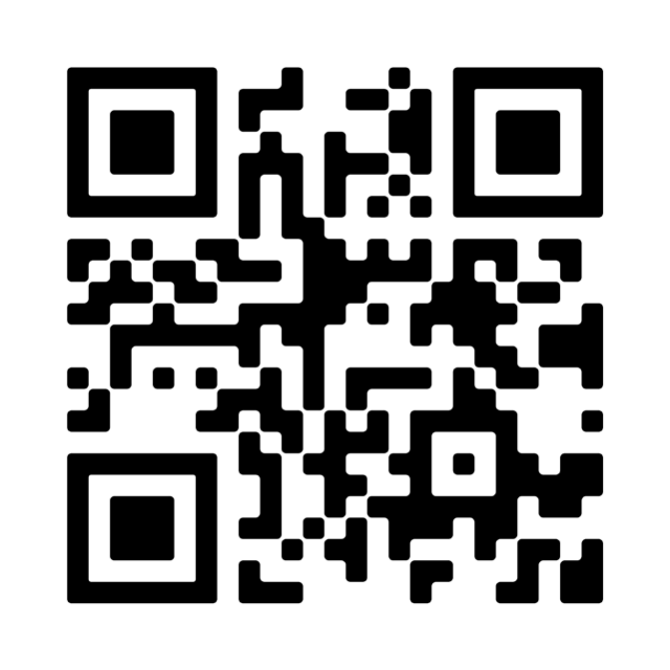# Isthmocele correction: resectoscopic, laparoscopic or both?

**DOI:** 10.52054/FVVO.15.3.086

**Published:** 2023-09-24

**Authors:** C Smet, B Nogueira, D Vilarinho, I Rodrigues, J Reis

**Affiliations:** Department of Obstetrics and Gynecology, Hospital de São Francisco Xavier, Lisboa, Portugal; Department of Obstetrics and Gynecology, Hospital da Luz Lisboa, Portugal

**Keywords:** Isthmocele, Laparoscopy, Hysteroscopy

## Abstract

**Background:**

An isthmocele is a myometrial defect in the site of the caesarean scar. In symptomatic women with abnormal uterine bleeding and secondary infertility, surgical correction can be considered. Most authors advocate that when there’s a residual myometrium ≥ 3mm it can be corrected through resectoscopic approach and when < 3mm the treatment should be laparoscopic, eventually guided by diagnostic hysteroscopy. Both these techniques have important limitations; therefore, the authors propose combining both techniques, in the same procedure, in order to overcome them.

**Objectives:**

To demonstrate the advantages of a surgical technique for correction of an isthmocele using both resectoscopic and laparoscopic resection.

**Materials and Methods:**

A stepwise demonstration of the technique with narrated video footage.

**Main Outcome Measures:**

Intraoperative data and outcomes in the patient’s follow-up.

**Results:**

One month after the surgery the patient was asymptomatic, reporting a resolution of the uterine abnormal bleeding, and the ultrasound showed a full correction of the isthmocele.

**Conclusion:**

A combination of resectoscopic and laparoscopic resection, in correcting bigger isthmoceles, is a good option to fully excise all the ﬁbrotic tissue.

**Learning Objective:**

This video aims to demonstrate the beneﬁts of using a technique combining resectoscopic and laparoscopic resection for correcting larger isthmoceles.

## Introduction

The isthmocele, a myometrial defect in the site of the caesarean scar, has been increasing with the raise in c-sections. Although usually asymptomatic, it can cause abnormal uterine bleeding and secondary infertility. Its diagnosis is made through ultrasound and it’s deﬁned as an isthmic indentation ≥2 mm (Jordans et al., 2019; Naji et al., 2012). In symptomatic women surgical correction can be considered (Gubbini et al., 2008). The majority of authors advocates that isthmoceles with a residual myometrium ≥ 2.5-3mm can be corrected through hysteroscopy and when < 2.5-3mm the treatment should be laparoscopic (Setúbal et al., 2020; Mashiach and Burke, 2021). Both these techniques have important limitations; the hysteroscopic resection is associated with a risk of bladder lesion, especially when the residual myometrium is thin, and in the laparoscopic approach the ﬁbrotic tissue localised in the lateral and posterior/deeper part of the isthmus can be difﬁcult to excise. Therefore, combining both techniques in the same procedure can overcome these limitations.

## Patients and methods

A 42-year-old patient was referred from an infertility clinic for an isthmocele correction. The patient presented abnormal uterine bleeding and secondary infertility, having had 3 unsuccessful embryo transfers. The ultrasound showed an isthmocele measuring 10x10 mm with a residual myometrium of 1 mm. After proper counselling the patient was proposed for a surgical correction of the caesarean defect. This video is a stepwise description of the technique using both resectoscopic and laparoscopic correction of the defect. Under general anaesthesia the procedure begins using a 15 Fr mini-resectoscope to remove the ﬁbrotic tissue on the lateral and posterior/ deeper part of the isthmocele. Then through laparoscopy we begin by performing a dissection of the paravesical fossa, followed by the dissection of the vesico-uterine space, in order to separate the bladder and visualise the isthmus. Using the 15 Fr mini-resectoscope, the dome of the isthmocele is identiﬁed with transillumination. A blunt cut of all the remaining anterior ﬁbrotic tissue is performed avoiding the use of energy, so as not to create more ﬁbrosis. The uterine wall is closed with a double layer suture, after inserting a Hegar probe in the cervical canal to prevent canal occlusion during suturing. The suture is done with simple separate stiches using a multiﬁlament absorbable surgical suture (VICRYL™ 1-0, polyglactin 910) with a ½ circle needle. The ﬁrst layer of separate stiches is performed deeper in the myometrium and the sutures are all knotted in the end to be certain not to grab the posterior wall of the uterus and inadvertently close the cervical canal. The second layer is more superﬁcial, closing the uterine serosa. When suturing, attention should be paid in restoring the normal anatomy, joining the superior and inferior parts of the scar at the same level. In the end the peritoneum is closed with an unlocked continuous single layer suture, using a monoﬁlament absorbable suture (MONOCRYL 2-0 - poliglecaprone 25). In larger isthmoceles, in addition to the measures mentioned above to prevent stenosis of the cervical canal, the authors perform a diagnostic hysteroscopy around 1-2 months after the procedure with eventual lysis of synechiae.

## Results

There were no surgical complications, and the patient was discharged in 24 hours. An ultrasound evaluation one month after the procedure showed a reconstructed myometrium and the patient referred a resolution of the uterine abnormal bleeding. The patient was advised to wait 6 months after the surgery before attempting a new pregnancy.

## Discussion

In larger isthmoceles the ﬁbrotic tissue is not only localized on the anterior part of the isthmus but seems to create an arch of fibrosis that extends through the lateral and posterior parts of the canal. The use of a mini-resectoscope requires minimum dilatation of the cervix which allows for a good visualization of the limits of the isthmocele. It is useful in resecting the ﬁbrotic tissue especially in the posterior part of the defect, which is difﬁcult to access through laparoscopy. In this way we can better restore the normal anatomy, preventing a deformation of the cervical canal. The anterior thinner part of the dome of the isthmocele is the one with higher risk of bladder lesion, thus being safer to excise through laparoscopy.

There is currently no robust evidence that one surgical method has better results in correcting an isthmocele and the associated symptoms (Zhang, 2016). Nonetheless, based on expert opinions, it has been proposed a hysteroscopic correction in smaller defects and a laparoscopic treatment in larger isthmoceles, with a cut-off of 2,5-3 mm (Setúbal et al., 2020; Mashiach and Burke, 2021). The combination of these two techniques seems to overcome the disadvantages of both.

When performing laparoscopic correction many use hysteroscopy as a guide to identify the place of the isthmocele, therefore, using the mini- resectoscope as both a way to identify the defect and to excise the more posterior ﬁbrotic tissue, will not increase the complexity of the surgery in terms of the necessary equipment.

## Conclusion

In symptomatic infertile women, a combination of resectoscopic and laparoscopic approach, in correcting larger isthmoceles, might be a good and safe option to fully excise the ﬁbrotic tissue. The combined advantages of both methods allow for a more thorough and complete correction of the defect. A bigger number of case reports as well as longer follow up are necessary to better deﬁne the advantages and possible disadvantages of this technique.

## Video scan (read QR)

https://vimeo.com/861216112/53f67f00d2?share=copy

**Figure:**